# Effects of rapid maxillary expansion in cleft patients resulting from the use of two different expanders

**DOI:** 10.1590/2177-6709.2016-001.aop

**Published:** 2016

**Authors:** Daniel Santos Fonseca Figueiredo, Lucas Cardinal, Flávia Uchôa Costa Bartolomeo, Juan Martin Palomo, Martinho Campolina Rebello Horta, Ildeu Andrade, Dauro Douglas Oliveira

**Affiliations:** 1 Former Orthodontic residents, Pontifícia Universidade Católica de Minas Gerais (PUC-MG), Belo Horizonte, Brazil.; 2 Associate Professor and Program Director, Case Western Reserve University, Department of Orthodontics, and Director of the Craniofacial Imaging Center, School of Dental Medicine, Cleveland, Ohio, USA.; 3 Associate Professor and Dean of Graduate Studies, Pontifícia Universidade Católica de Minas Gerais (PUC-MG), Belo Horizonte, Brazil.; 4 Associate Professor of Orthodontics, Pontifícia Universidade Católica de Minas Gerais (PUC-MG), Belo Horizonte, Brazil.; 5 Associate Professor and Program Director of Orthodontics, Pontifícia Universidade Católica de Minas Gerais (PUC-MG), Belo Horizonte, Brazil.

**Keywords:** Palatal expansion technique, Cleft palate, Cone-beam computed tomography

## Abstract

**Objective:**

The aim of this study was to evaluate the skeletal and dental effects of rapid maxillary expansion (RME) in cleft patients using two types of expanders.

**Methods:**

Twenty unilateral cleft lip and palate patients were randomly divided into two groups, according to the type of expander used: (I) modified Hyrax and (II) inverted Mini-Hyrax. A pretreatment cone-beam computed tomographic image (T0) was taken as part of the initial orthodontic records and three months after RME, for bone graft planning (T1).

**Results:**

In general, there was no significant difference among groups (*p* > 0.05). Both showed a significant transverse maxillary expansion (*p* < 0.05) and no significant forward and/or downward movement of the maxilla (*p* > 0.05). There was greater dental crown than apical expansion. Maxillary posterior expansion tended to be larger than anterior opening (*p* < 0.05). Cleft and non-cleft sides were symmetrically expanded and there was no difference in dental tipping between both sides (*p* > 0.05).

**Conclusions:**

The appliances tested are effective in the transverse expansion of the maxilla. However, these appliances should be better indicated to cleft cases also presenting posterior transverse discrepancy, since there was greater expansion in the posterior maxillary region than in the anterior one.

## RESUMO

**Objetivo:**

o objetivo deste estudo foi avaliar os efeitos esqueléticos e dentários da expansão rápida da maxila (ERM) em pacientes fissurados, utilizando dois tipos de disjuntores.

**Métodos:**

vinte pacientes com fissura labiopalatal unilateral foram aleatoriamente divididos em dois grupos, de acordo com o tipo de aparelho utilizado: (1) Hyrax modificado e (2) Mini-Hyrax invertido. Tomografias computadorizadas de feixe cônico foram obtidas antes do tratamento (T0), como parte da documentação ortodôntica inicial, e três meses após a ERM, para o planejamento de enxertia óssea (T1).

**Resultados:**

não houve diferença significativa entre os grupos (*p*>0,05). Ambos apresentaram significativa expansão transversal da maxila (*p*<0,05), sem significativa movimentação anterior e/ou inferior da maxila (*p*>0,05). Houve uma maior expansão transversal das coroas em relação à expansão nos ápices. A tendência observada foi uma maior expansão na região posterior da maxila, em comparação à anterior (*p*<0,05). Avaliando o deslocamento dos lados fissurado e não fissurado, a expansão ocorreu de maneira simétrica e não houve diferença na inclinação dentária entre os lados (*p*>0,05).

**Conclusões:**

os aparelhos testados são eficazes na expansão transversal da maxila em pacientes fissurados. Porém, esses aparelhos seriam melhor indicados para casos de fissura labiopalatal com atresia transversal posterior, uma vez que a expansão foi maior na região posterior da maxila do que na região anterior.

## INTRODUCTION

Cleft lip and palate (CLP) is a relatively common birth defect that affects the craniofacial complex.^1^ ^,^ ^2^ During the fi st years of life, CLP patients are subjected to primary repair surgeries. As a consequence, the scar tissue compromises growth and development of the maxilla while frequently causing maxillary constriction. Therefore, rapid maxillary expansion (RME) is a therapy commonly used to correct this transverse deficiency.^3^ ^,^ ^4^

RME effects in non-cleft patients is well documented in the literature.^5^ ^-^ ^15^ However, the biomechanical effects of RME in CLP patients seem to be different from those registered for patients without this craniofacial deformity, probably due to different anatomical structures.16,17 This high anatomical variability in the maxillary arch has led to the development of maxillary expanders with alternative designs.^4^ ^,^ ^17^ ^,^ ^18^ ^,^ ^19^ A recent study evaluated the effects of expanders designed to privilege anterior arch expansion: the fan-type and inverted mini-Hyrax (iMini) associated with a transpalatal arch (TPA).^17^ However, the effects of the iMini without the TPA were not addressed. Therefore, the aim of the present study was to evaluate and compare the dentoskeletal effects of modified Hyrax and iMini supported on first permanent molars.

## MATERIAL AND METHODS

The study sample consisted of 20 unilateral cleft lip and palate (UCLP) children (14 boys, 6 girls) who sought orthodontic treatment at the Center of Craniofacial Anomalies (CENTRARE), Department of Orthodontics, Pontifícia Universidade Católica de Minas Gerais. The selection criteria were: presence of UCLP, need for maxillary expansion treatment and age between 8 and 15 years. Exclusion criteria included: absence of maxillary fi st molars, periodontal disease, previous orthodontic treatment and presence of any syndrome. Cervical vertebral maturation revealed that all patients were before or during the pubertal growth spurt (cervical maturation between CS1 to CS4).^20^ This study was approved by the local Ethics Committee, and an informed consent form was obtained from all patients’ parents.

**Figure 1:**
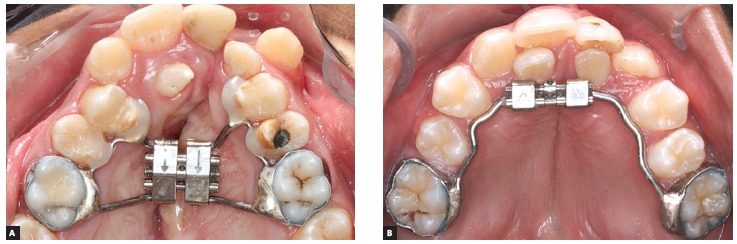
Rapid maxillary expanders evaluated: A) modified Hyrax; B) inverted mini-Hyrax (iMini).

The sample was randomly allocated into two groups with 10 patients each: (1) modified Hyrax expander and (2) iMini supported on first permanent molars. Sex and age distributions are shown in Table 1 for all groups. The modified Hyrax is a tooth-borne appliance (Leone, Florence, Italy) with a jackscrew placed in the region of deciduous molars or premolars (Fig 1A). The iMini is a tooth-borne appliance (Dynaflex, Sait Ann, Missouri, USA) designed with a mini-screw positioned at the anterior region (Fig 1B). All expanders were made by the same technician, and the bands were placed only on maxillary first molars with wire extensions bonded to the adjacent teeth.

The methods were similar to those used in our previous study.^17^ A pretreatment cone-beam computed tomographic image (CBCT) (T0) was taken as part of the initial orthodontic records of all patients. The activation regimen was established at two turns/day until the tip of the lingual cusp of maxillary teeth touched the tip of the buccal cusp of mandibular teeth. The appliance was kept in place as a passive retainer for three months. Aft r the retention period, the expander was removed and a post expansion CBCT image (T1) was immediately taken. On the same day, a transpalatal bar with anterior extensions was inserted as a retainer. The T1 CBCT was justified because of its valuable importance in bone graft planning. None of the patients received any brackets or wires in the maxillary arch until the second CBCT image was taken.

All scans were obtained by the same technician with an i-CAT machine (Imaging Sciences International, Hatfield, Pa, USA), performed at 120 kV, 8 mA, scan time of 40 seconds, and 0.3-mm voxel dimension. All CBTC images were oriented and standardized by means of Dolphin Imaging software (version 11.5, Dolphin Imaging & Management Solutions, Chatsworth, Calif, USA). Patient’s head was oriented in the three planes of space for frontal, right lateral and top (facing down) views, as detailed previously.^17^

**Figure 2:**
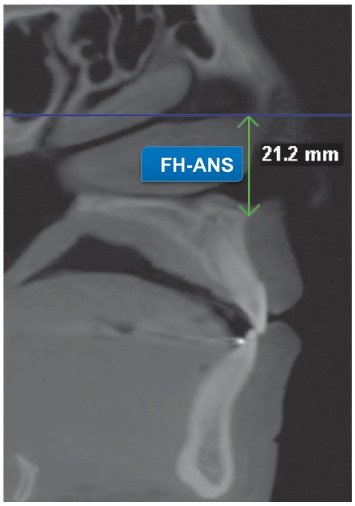
Vertical measurement (FH-ANS).

To examine the effects of RME, the measurements were evaluated at T0 and T1 in three planes of space: anteroposterior (AP), vertical and transversal. The AP plane was assessed in lateral cephalograms obtained through CBCT by the SNA measurement. The vertical plane was verified by means of CBCT sagittal slices, measuring the smaller distance between the Frankfort Horizontal Line and ANS (FH-ANS) (Fig 2).

**Figure 3:**
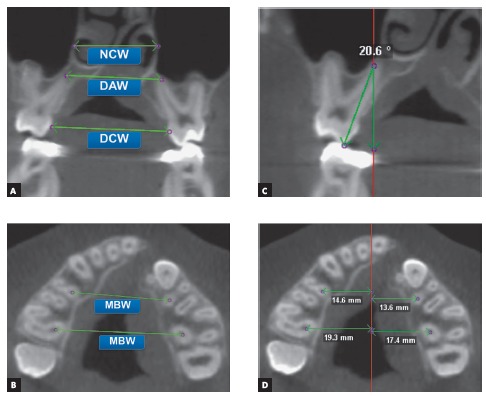
Transversal measurements were performed in the anterior and posterior regions of the maxilla. A) Dental crown width (DCW), dental apices width (DAW), nasal cavity width (NCW) measurements. B) Anterior and posterior MBW measurements. C) Coronal slice showing dental tipping. D) Lateral displacement between cleft and non-cleft sides.

Transverse changes were measured in the anterior and posterior regions of the maxilla. Transverse posterior maxillary measurements were taken at the level of the first permanent molars. Transverse anterior measurements were taken at the level of the most anterior appliance-supporting teeth. As described previously,17 the following parameters were used to quantify the amount of transversal expansion (Figs 3A, 3B and 3C): dental crown width (DCW), maxillary basal width (MBW), dental apices width (DAW), nasal cavity width (NCW), and dental tipping (Tip).

To evaluate which maxillary segment was more expanded, a mid-sagittal line connecting the Crista Galli and Basion was defined as the reference line. In the axial slice, the smaller distance from this mid-sagittal line to the four MBW landmarks was measured (Fig 3D).

### Statistical analysis

All measurements were performed by the same operator blinded to group status. In order to test intraexaminer reproducibility, 18 random images were remeasured by the same examiner, with at least one week between them, and compared to the original measurements. Intraexaminer reliability values were determined with the intraclass correlation coefficient. Chisquare test was performed to verify the distribution of the cleft-side as well as of patient’s sex between groups. Paired *t*-test was used to evaluate whether the changes from T0 to T1 were significantly different in each group. Unpaired *t*-test was performed to statistically compare the patients’ age between the two groups and to evaluate differences in the changes of each measurement between the different appliances. Data obtained from all measurements were processed with GraphPad Prism (version 5.01, GraphPad Software, San Diego, Calif, USA). The level of significance for all statistical tests was predetermined at 5%. Intraexaminer reproducibility test varied between 0.98 and 0.99, indicating high reproducibility among measurements.

**Table 1:**
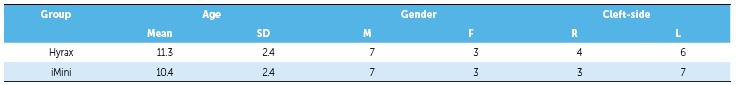
Distribution of age (years), sex and cleft-side.

Unpaired t-test showed no statistically difference between groups age (p=0.452); the chi-square test showed no statistically difference between groups for gender (p=1.000) and cleft-side (p=0.639) distribution.

## RESULTS

There was no significant forward and/or downward movement of the maxilla in either one of the groups. As shown in Tables 2 and 3, there was no statistically significant maxillary movement in the vertical or anteroposterior planes (*p* > 0.05), and there was no difference between groups for this measurement (*p* > 0.05) (Table 4). There was significant transverse maxillary expansion in both groups, and no significant difference was found between them. All linear parameters observed in the transverse maxillary dimensions demonstrated significant difference in both groups (*p* < 0.05), including NCW, as shown in Tables 2 and 3. In comparing both groups, there were no differences in any measurement studied (*p* > 0.05) (Table 4).

Both groups showed greater dental crown than apical expansion. Measurements (Tables 2 and 3) indicated that the greatest widening occurred in the crown area, and that the widening effect of the device gradually decreased throughout the upper structures.

Maxillary posterior expansion tended to be larger than anterior opening in both groups. When comparing the means of difference between anterior and posterior regions within the same group, most variables showed greater posterior than anterior expansion (*p* < 0.05) (Table 5), except for NCW in both groups and for the variable DCW in the Hyrax group (*p* > 0.05).

There was no significant difference in dental tipping between appliances. There were no statistically significant differences in anterior or posterior dental tipping when the two appliances were compared (*p* > 0.05) (Table 4). Additionally, it was perceived that both groups demonstrated greater anterior than posterior dental tipping.

**Table 2:**
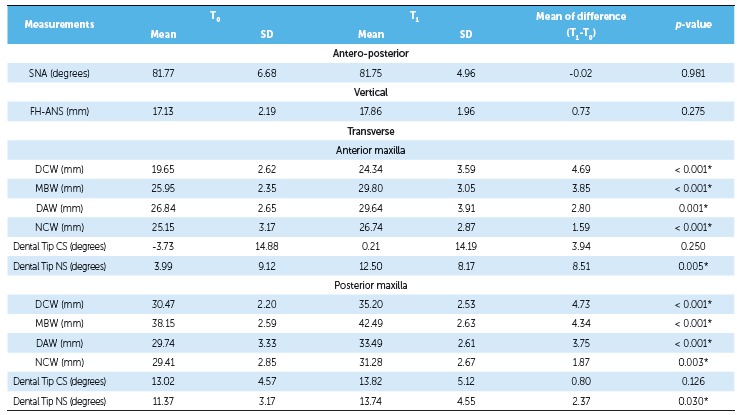
Comparison between T and T maxillary dimensions in the Hyrax group.

*p* values were obtained by paired *t*-test; *statistically significant *p* value; SD = standard deviation; CS = cleft side; NS = non-cleft side.

**Table 3:**
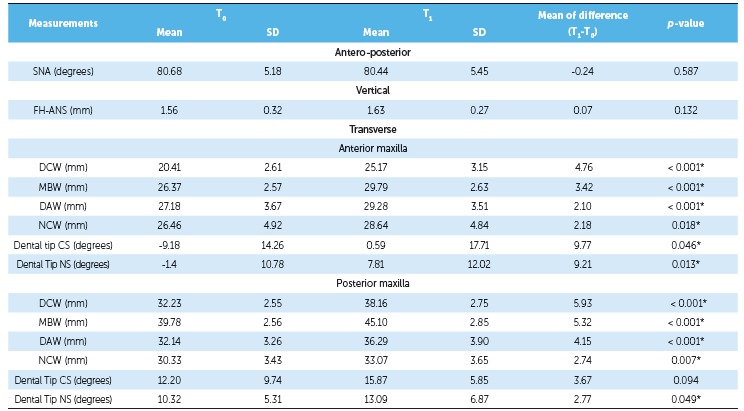
Comparison between T and T maxillary dimensions in the iMini group.

*p*-values were obtained by paired *t* test; *statistically significant *p*-value; SD = standard deviation; CS = cleft side; NS = noncleft side.

**Table 4:**
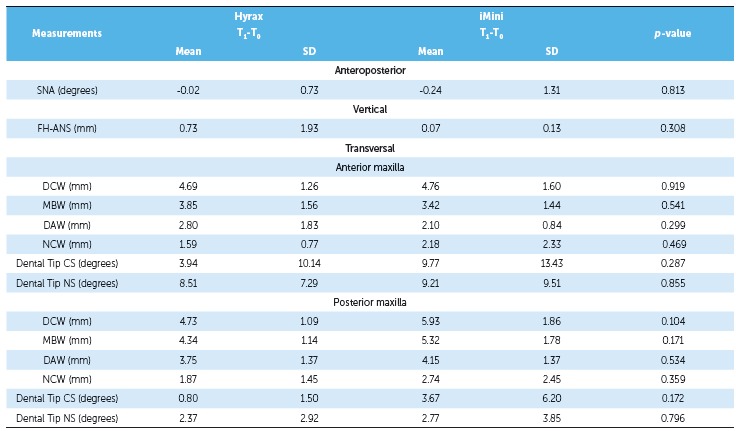
Comparisons between the changes of both groups.

*p*-values were obtained by unpaired *t* test; *statistically significant *p*-value; SD = standard deviation; CS = cleft side; NS= noncleft side.

**Table 5:**
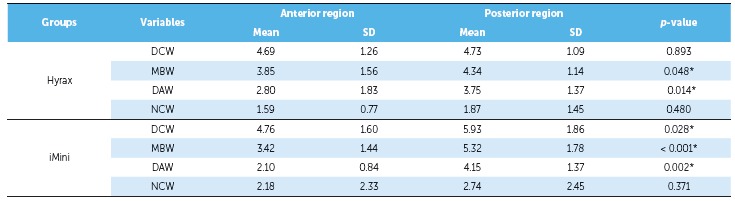
Transverse changes (mm) comparison between anterior and posterior region for each expander.

*p*-values were obtained by paired *t* test; *statistically significant *p*-value; SD = standard deviation.

Cleft and non-cleft sides were symmetrically expanded and there was no difference in dental tipping between groups. There was no significant difference in the amount of expansion when cleft and non-cleft sides were compared in each group (*p* > 0.05) (Table 6). When the 20 patients were evaluated together, still there was no significant difference between cleft and non-cleft sides (*p* > 0.05) (Table 6). There was also no difference in dental tipping between the cleft side and the noncleft side (*p* > 0.05) (Table 7).

## DISCUSSION

Despite being a widely used procedure in patients with CLP, RME treatment-related structural changes in these patients have only been evaluated by a small number of studies.^17^ ^,^ ^21^ ^,^ ^22^ ^,^ ^23^ A previous study in cleft patients using CBCT evaluated the effects of expanders developed to focus on expansion of the anterior region of the arch.^17^ It was shown that fan-type and iMini expanders - both anchored in premolars associated with TPA - were effective in expanding the anterior region, thus restricting the posterior expansion.^17^ ^,^ ^19^ By using similar methods and evaluating the same variables, the objective of this study was to evaluate and compare the dentoskeletal effects of RME in cleft patients using the modified Hyrax expander and iMini anchored in first permanent molars without TPA.

**Table 6:**
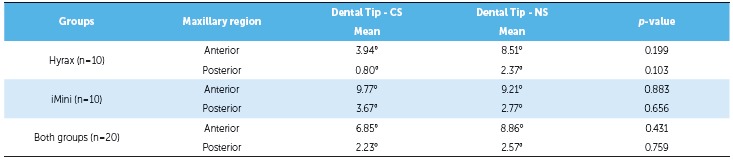
Dental tipping on cleft side and noncleft side.

*p*-values were obtained by paired *t* test; *statistically significant *p*-value; SD = standard deviation; CS = cleft side; NS = noncleft side.

**Table 7:**
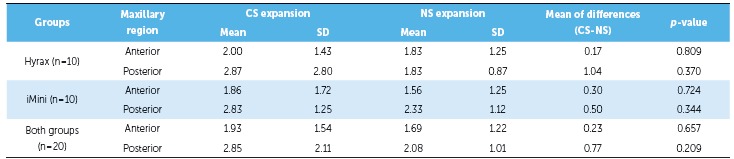
Alveolar expansion (mm) on cleft side and noncleft side.

*p*-values were obtained by paired *t* test; *statistically significant *p*-value; SD = standard deviation; CS = cleft side; NS = noncleft side.

The present study had some important features: it was a prospective study; patients were randomly divided between groups, and skeletal maturation was assessed. All sample subjects were treated when they were at the cervical maturation stage between CS1 and CS4. There was no untreated control group due to ethical concerns and short treatment time.

The iMini and modified Hyrax groups revealed no significant forward or downward movement of the maxilla. There were discordant results of studies with noncleft patients which described significant forward^11^ ^,^ ^12^ ^,^ ^13^ ^,^ ^24^ and downward^11^ ^,^ ^12^ ^,^ ^14^ ^,^ ^15^ ^,^ ^24^ displacement. However, previous studies with CLP patients also showed no change in anteroposterior plane aft r RME.^17^ ^,^ ^23^ Thus, these fi dings suggest that the differential anatomy in cleft patients, in comparison to non-cleft ones, can induce to a different behavior of the maxilla in the sagittal and vertical planes.^17^

 All linear parameters observed in the transverse dimension presented significant changes for both appliances, indicating that both are effective in performing RME. As in previous RME studies,^7^ ^,^ ^9^ ^,^ ^10^ ^,^ ^14^ ^,^ ^25^ the present fi dings indicated that the greatest widening occurred in the dentoalveolar area, and the widening effect of the device gradually decreased throughout the upper structures in a triangular pattern, indicating that dental overexpansion is necessary to gain the appropriate skeletal effect.

CLP patients most commonly present atresia in the anterior maxillary region.^3^ ^,^ ^4^ ^,^ ^26^ Thus, posterior expansion may be undesirable in certain cases because the posterior limit of expansion can be reached before the desired anterior expansion is obtained. From this perspective, the present results showed a pattern of unfavorable opening when using both devices. Maxillary posterior expansion tended to be larger than anterior opening in both groups. There was a previous expectation that iMini would achieve greater expansion in the anterior maxilla because of the anterior location of the screw. The resultant force would be located more distant from the center of resistance of each maxillary half,^27^ which would theoretically propitiate more expansion in the anterior region rather than in the posterior region. However, this expectation was not confirmed. Therefore, in order to prioritize expansion in the anterior region, it would be important to consider the association of a TPA with iMini or the use of a fan-type expander, as suggested by previous articles.^17^ ^,^ ^19^ Thus, it is believed that some patients in this study would have more effective maxillary expansion if they were treated with these devices;^17^ ^,^ ^19^ however, at the time they were treated, the effectiveness of these devices had not been evinced yet.

Considering dental tipping, both groups demonstrated greater anterior than posterior dental tipping. This would be expected, since posterior supporting teeth were banded and firmly attached to the appliance, whereas anterior supporting teeth were just connected by lingual wire extension. As the screw was activated, the bands provided resistance to tipping, which probably led to a greater bodily buccal movement of the banded teeth compared to non-banded teeth.^5^

Previous studies have shown an association between RME and various degrees of increase in nasal cavity dimension.^9^ ^,^ ^11^ ^,^ ^14^ ^,^ ^25^ Ptresent data clearly showed that both groups demonstrated an increase at the posterior and anterior regions in nasal cavity width, and there was no significant difference when the two groups were compared.

Due to an asymmetrical anatomy of the maxilla, some studies have evaluated if the cleft and non-cleft sides of the maxilla are symmetrically expanded.^16^ ^,^ ^17^ ^,^ ^22^ Our findings showed a symmetrical expansion in both groups, thereby confirming previous results.^17^ When all 20 patients were evaluated together, still there were no significant differences between cleft and non-cleft sides. Furthermore, there was no significant difference in dental tipping in the cleft side when compared with the non-cleft side.

Despite showing similar dentoskeletal results, the Hyrax expander presents a greater size, volume and extent than iMini. Therefore, iMini, as described herein and in previous articles,^17^ ^,^ ^19^ may be a good alternative expander to minimize the difficulty in maintaining appropriate oral hygiene during RME. Thus, the use of this more delicate expander may reduce the negative impact of orthodontic treatment in cleft patients. However, future studies evaluating the impact of these appliances on the quality of life of cleft patients are necessary to confirm this hypothesis.

## CONCLUSIONS

Based on this clinical trial, the following conclusions can be drawn:

» There was no significant anteroposterior or vertical movement of the maxilla with RME.
» RME produced significant increases in all linear measurements of the maxillary transverse dimension for both groups, including nasal cavity.
»The cleft side and the non-cleft side expanded symmetrically.
» The tested appliances were effective in maxillary expansion. However, these appliances should be better indicated to cleft cases also presenting posterior transverse discrepancy, since there was greater expansion in the posterior maxillary region in comparison to the anterior region.
